# Impact of breast cancer surgery on angiogenesis circulating biomarkers: a prospective longitudinal study

**DOI:** 10.1186/1477-7819-11-213

**Published:** 2013-08-27

**Authors:** Georgios K Georgiou, Maria Igglezou, Ioannis Sainis, Katerina Vareli, Haralampos Batsis, Evangelos Briasoulis, Michalis Fatouros

**Affiliations:** 1 Department of Surgery, University Hospital of Ioannina, Stavros Niarchos avenue, Ioannina 45500, Greece; 2 Cancer Biobank Center, University of Ioannina, Stavros Niarchos avenue, Ioannina 45500, Greece; 3 Department of Biological Application and Technologies, University of Ioannina, Stavros Niarchos avenue, Ioannina 45500, Greece

**Keywords:** Angiogenesis, Breast cancer, Transcripts, PCR arrays, ELISA

## Abstract

**Background:**

Debate about the potential effects that surgery might have on cancer cells dormancy and angiogenesis prompted us to investigate the impact of breast surgery on circulating angiogenesis modulating gene transcripts and proteins.

**Methods:**

Blood samples from 10 female patients diagnosed with breast cancer and 6 with fibroadenoma were collected before surgery and post-operatively on days 3 and 7 (breast cancer patients only). A set of 84 angiogenesis-associated transcripts were assessed using quantitative PCR arrays, and circulating protein levels (vascular endothelial growth factor A (VEGFA), IL8 and fibroblast growth factor 2 (FGF2) were measured using ELISA in the same samples. The results were investigated against clinicopathological data and patient outcome.

**Results:**

Plasma levels of VEGFA and IL8 after surgery were significantly elevated in the breast cancer group compared to the control group (*P =* 0.038 and *P* = 0.021, respectively). In the cohort of breast cancer patients, VEGFA increased on day 3 (*P* = 0.038) and declined on day 7 (*P*= 0.017), while IL8 did not change on day 3 but showed a significant decline on day 7 (*P* = 0.02). FGF2 levels did not change significantly over time. Regarding gene transcripts, we detected upregulation of a significant number of angiogenesis-specific genes in patients with breast cancer versus controls: sphingosine kinase 1(SPHK1), epidermal growth factor (EGF), vascular endothelial growth factor C (VEGFC), neuropilin 1 (NRP1), fibroblast growth factor (FGF1), laminin alpha 5 (LAMA5), collagen type IV alpha 3 (COL4A3), IL8, ephrin B2 (EFNB2), ephrin A3 (EFNA3), tyrosine endothelial kinase (TEK), integrin beta 3 (ITGB3), AKT1, thrombospondin 1 (THBS1), chemokine (C-C motif) ligand 11 (CCL11) and TIMP metallopeptidase inhibitor 3 (TIMP3). Surgery induced an altered expression in several keygenes in breast cancer patients. We identified an upregulation of COL4A3 and downregulation of chemokine (C-X-C motif) ligand 9 (CXCL9), EGF, FGF1, Kinase insert domain receptor (KDR), Placental growth factor (PGF), TIMP3 and VEGFC.

**Conclusion:**

Breast cancer patients have a different expression profile of circulating angiogenesis biomarkers compared to patients with fibroadenoma. Moreover, mastectomy promotes a transient increase of VEGFA and a shift in the expression patterns of a broad panel of angiogenesis-related circulating gene transcripts.

## Background

Surgery is the cornerstone of therapy for early breast cancer. Resection of the primary tumor and axillary lymph nodes in the absence of distant metastases has always been considered curative for non-metastatic disease. However, development of distant metastases within the first few years after tumor resection challenges this notion to some extent [1,2]. A number of experimental and clinical studies have suggested that, although surgical resection of breast cancer has beneficial effects for the majority of patients, it might also trigger cancer spread and growth in some others [3,4].

It is now known that breast cancer, even in the early stages, is systematically spread in one-third of the patients already with a diagnosis [5]. It is therefore thought that surgery might affect cancer cell dormancy and trigger angiogenesis [6-9]. Currently, several studies have focused on angiogenesis in breast cancer [10,11] but only a few have investigated the kinetics of the best known circulating pro-angiogenic molecules in blood or fluid from wound drains and their expression in cancer tissue [12-15].

We studied the expression of a set of 84 circulating angiogenesis-regulating gene transcripts and key proteins in patients with breast cancer and evaluated their expression pattern and kinetics following mastectomy for potential clinical relevance.

## Methods

### Patients

Ten breast cancer patients and six patients with fibroadenoma were enrolled in this study. The Institutional Review Board of the University of Ioannina approved the study protocol. Inclusion criteria were scheduled surgery for operable early breast cancer or benign tumor and patient consent. Exclusion criteria were the following: diabetes mellitus, active inflammation during the peri-operative period, recent myocardial infarction, peri-operative blood transfusion, erythropoietin administration, and synchronous malignancies because of potential interference with angiogenesis [16]. A signed informed consent was obtained from all participating in the study patients.

Patient demographics are shown in Table 1. The six patients with benign breast disease initially underwent an excisional biopsy in macroscopically healthy borders due to suspicious mammographic findings. If frozen section microscopy excluded cancer, a suction drain was placed and the operation terminated. All breast cancer patients had a core-biopsy performed prior to surgery. The type of procedure (modified radical mastectomy or wide local excision) was decided prior to surgery, following surgeon consultation and considering the patient’s desire. The same experienced Senior Surgeon who leads the Breast Unit performed all surgical procedures. A standard protocol of general anesthesia was applied to all cases, using Propofol–Fentanyl–muscle relaxant induction and O_2_–Sevoflurane maintenance. Histopathologic examination of specimens was done by a single pathologist who specializes in breast tissue biopsies and immunohistochemistry. Patients with positive axillary lymph nodes received adjuvant therapy according to tumor biology, while those that had wide local excision also received adjuvant thoracic radiotherapy.

**Table 1:** Demographics

	**Group A (benign breast disease)**	**Group B (breast cancer patients)**
Number of patients	6	10
Median age (years)	37 (range, 28–47)	55 (range, 36–76)
Disease	Fibroadenoma	Invasive ductal carcinoma
**Disease site**		
Right breast	3/6	8/10
Left breast	3/6	2/10
**Surgical procedure**		
WLE	6/6	-
WLE + ALND	-	5/10
MRM + ALND	-	5/10
**T stage**		
T_1_	-	0/10
T_2_	-	5/10
T_3_	-	5/10
**N status**		
N_0_	-	6/10
N_1_	-	3/10
N_2_	-	1/10
**Grade**		
2	-	9/10
3	-	1/10
**Hormonal receptor status**		
ER+	-	10/10
PR+	-	10/10
**HER2 status**		
0	-	4/10
1	-	2/10
2	-	4/10
**Blood samples**	PRE, day 3	PRE, day 3, day 7

*ALND* axillary lymph node dissection, *ER* estrogen receptors, *MRM* modified radical mastectomy, *PR* progesterone receptors, *PRE* day before surgery, *WLE* wide local excision.

### Acquisition of blood samples and storage

Blood was drawn via venipuncture by the same person every time, using a 21G BD Vacutainer® Safety-Lok™ Blood Collection Set (BD Diagnostics, Franklin Lakes NJ, USA). For each patient with breast cancer, three blood collections were undertaken at the following time intervals: on the day before surgery and on postoperative days 3 and 7. Patients with fibroadenoma had their blood samples collected preoperatively and on day 3 after surgery. All samples were collected in the morning (between 8 and 10 am) and at room temperature. Each blood collection consisted of two PAXgene™ Blood RNA tubes (PreAnalytix, GmbH, Hombrechtikon, Switzerland), containing 2.5 ml whole blood each (plus 6.9 ml RNA stabilization reagent) and four plasma BD Vacutainer® tubes (BD Diagnostics), containing 2 ml whole blood (together with K_2_ethylenediaminetetraacetic acid (EDTA) at a concentration of 3.6 mg/ml).

PAXgene™ Blood RNA tubes were carefully incubated 8 to 10 times for appropriate mixing with the reagent and then placed in the upright position at room temperature for at least 2 hours (according to the manufacturer’s suggestions), before being finally stored at −80°C. Plasma samples were centrifuged for 15 minutes at 3,000 rpm (at room temperature) within 30 minutes of the collection time. The supernatant was collected with a pipette and placed in Eppendorf® (Eppendorf-Netheler-Hinz, GmbH, Hamburg, Germany) tubes before freezing at −80°C. All samples were stored frozen at the University of Ioannina Cancer Biobank Center. Collection of all of the samples had been completed within a period of 2 months.

### RNA isolation

For RNA extraction, the PAXgene blood RNA extraction kit (PreAnalytix, GmbH) was used according to the manufacturer’s instructions. RNA integrity was checked by RNA electrophoresis. Only RNAs with RNA integrity number >7 were used for reverse transcription and further processing. Purified RNA samples were then subjected to quantitative analysis, using the NanoDrop® ND-1000 spectophotometer (Thermo Fisher Scientific®, Wilmington, DE, USA), with measurements in a range of 230 to 350 nm. A_260_/A_280_ values were about 2 for all samples, indicating highly purified RNA. Qualitative analysis was performed with RNA gel electrophoresis 1.2%. For reverse transcription, the SABiosciences RT^2^ First Strand kit was used (SABiosciences/Qiagen, Frederick, MA, USA). In all cases 0.5 μg RNA were reversed transcribed. Simultaneous quantification of 84 gene transcripts involved in angiogenesis was performedusing the angiogenesis RT^2^ profiler PCR Array (PAHS-024F, SABiosciences/Qiagen). Relative expression was determined with the LightCycler® 480 instrument (Roche, Rotkreuz, Switzerland) and the ΔΔCt method [17].

### Vascular endothelial growth factor A, fibroblast growth factor 2 and interleukin 8 protein measurements

EDTA plasma samples were analyzed using commercial ELISA kits for vascular endothelial growth factor A (VEGFA), fibroblast growth factor 2 (FGF2) and CXCL8/IL8 (R&D Systems Inc, Quantikine, Minneapolis, MN, USA). Aliquots of 100 μl were used for VEGFA and FGF2 and 50 μl for IL8 analysis. All analyses and calibrations were carried out in duplicate. The calibration on each microplate used recombinant VEGFA, FGF2 and IL8 standards, respectively. Optical densities were determined using a microplate reader at 450 nm. The blank was subtracted from the duplicate readings for each standard and sample. A standard curve was created by plotting the logarithm of the mean absorbance of each standard versus the logarithm of the VEGFA, FGF2 and IL8 concentrations. VEGFA, FGF2 and IL8 concentrations are reported as pg/ml. The sensitivity of the assay for VEGFA is 9 pg/ml with a detection range of 31.2 to 2,000 pg/ml. The ELISA kit for CXCL8/IL8 has a sensitivity of 1.5 to 7.5 pg/ml and the reliable standard curve ranges from 31.2 to 2,000 pg/ml. The Quantikine® Human FGF2 immunoassay kit has a minimum detectable dose less than 3 pg/ml with a detection range of 10 to 640 pg/ml.

### Patient outcome

All patients were assessed for local recurrence or development of metastatic disease according to standard follow-up protocols. Duration of follow-up ranged from 25 to 37 months (mean, 31 months). During follow-up none of the patients died but one patient (aged 58 years) who had undergone a wide local excision due to a T_2_N_2_ invasive ductal carcinoma was re-operated because of local recurrence in the axilla 3 years later.

### Statistical analysis

Paired and unpaired *t* tests were performed using GraphPad Prism version 6.00 for Mac OS X (GraphPad Software, La Jolla, CA, USA; http://www.graphpad.com). Differences with *P*< 0.05 were considered statistically significant.

## Results

### Plasma levels of circulating pro-angiogenic factors

Firstly, we measured plasma levels of VEGFA, IL8 and FGF2, three of the most significant pro-angiogenic factors.

**Figure 1:**
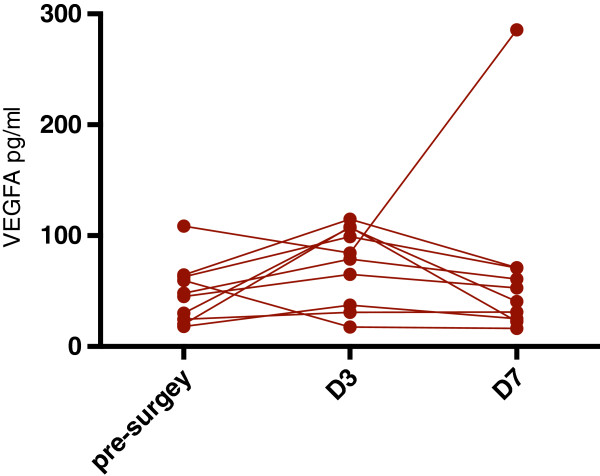
**Aligned plot of repeated measures of vascular endothelial growth factor A (VEGFA) with matched values stacked in subcolumns.** The outlier refers to a patient with locally advanced disease (T_2_N_2_) who eventually relapsed.

**Figure 2:**
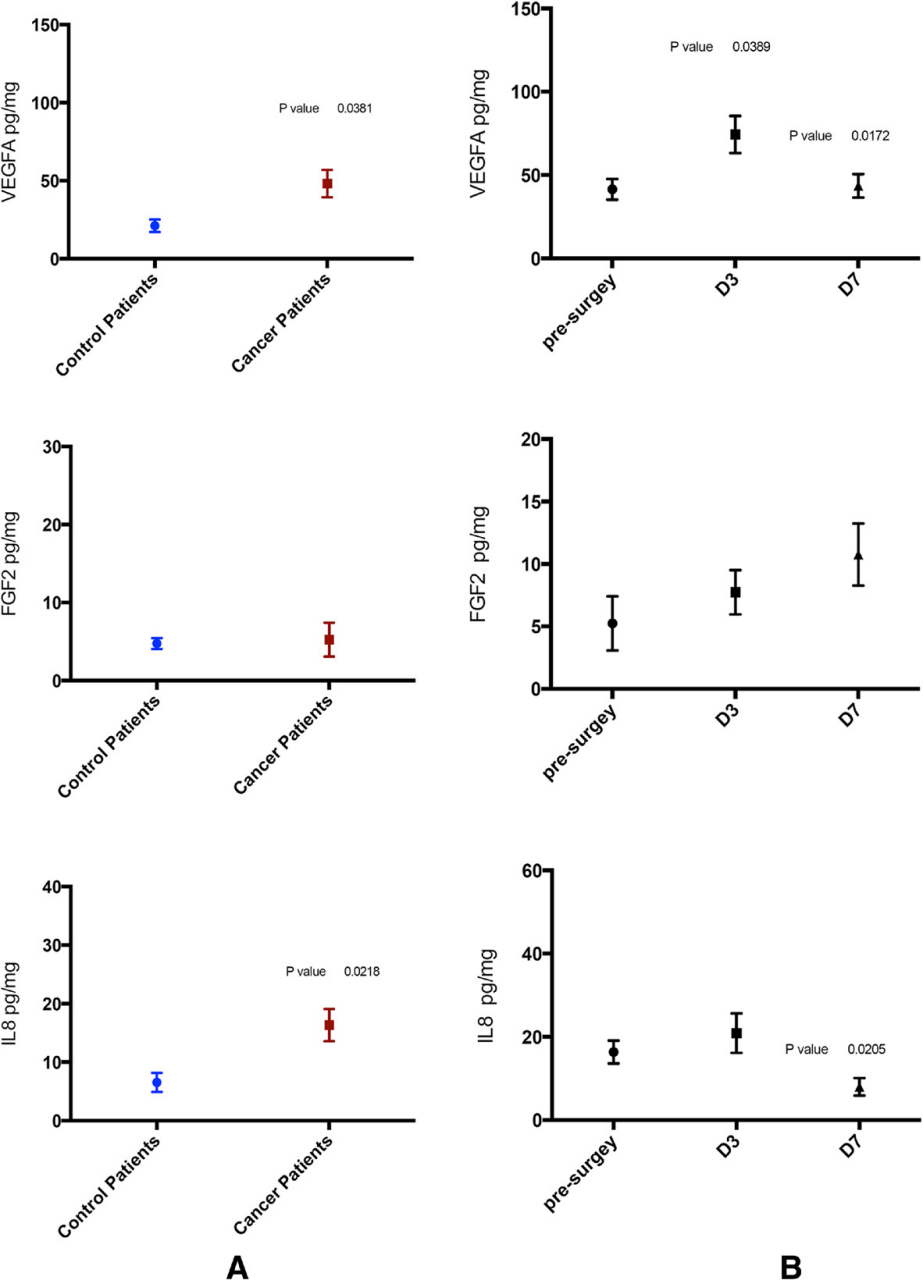
**Circulating vascular endothelial growth factor A (VEGFA), fibroblast growth factor 2 (FGF2) and IL8 levels measured at different time points before and after surgery. (A)** Both groups of patients. **(B)** Breast cancer patients only**.** Values are shown as means± SEM. D3,sampling on postoperative day 3; D7,sampling on postoperative day 7. Only significant *P* values are shown and refer to paired *t* tests.

Regarding VEGFA, non-cancer subjects had values ranging between 10 and 36.9 pg/ml preoperatively (mean, 21.145 pg/ml), in contrast to breast cancer patients who showed values between 18 and 108.54 pg/ml (mean, 48.17 pg/ml), a difference which proved to be statistically significant (*P* = 0.038*)*. In the group of breast cancer patients, VEGFA initially increased in all patients and declined back on day 7, except for one outlier with locally advanced disease (T_2_N_2_) who eventually relapsed (Figure 1). These changes reached a statistical significance on day 3 compared to presurgical values and on day 7 compared to day 3 (*P* =0.038 and 0.017, respectively; Figure 2B).

FGF2 values were quite similar for both the control and the study group preoperatively and in some cases below the detection limit of the assay. A postsurgical increase was documented in most patients. This increase continued on day 7 following surgery for the breast cancer group. However, these changes did not reach statistical significance at any time point (Figure 2).

IL8 expression was higher in the study group prior to surgery, with values ranging between 2.59 and 33.7 pg/ml (mean, 16.33 pg/ml), a difference that was statistically significant (*P* = 0.021)*.* In breast cancer patients, IL8 initially showed a slight increase towards day 3, but on day 7 it decreased to levels lower than the preoperative values (*P* = 0.02) (Figure 2).

### Gene expression analysis

**Figure 3:**
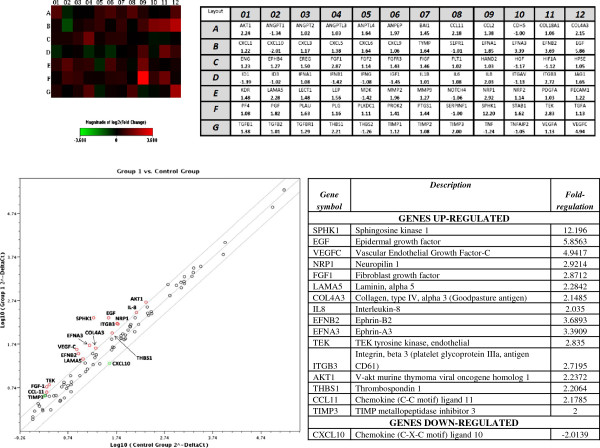
**Scatter plot analysis and heat map of all patient samples preoperatively.** The heat map demonstrates comparative expression of all 84 genes. Significantly upregulated genes (for example, SPHK1) are depicted in a more intense red color (F09), while significantly downregulated genes are shown with a more intense green color (for example, CXCL10 in B02). The graph below plots the log_10_ of normalized gene expression levels between the control group (*x*-axis) and the breast cancer group (*y*-axis). Genes with a more than twofold upregulation in the breast cancer group are depicted in the upper left corner with red dots, while genes which are more than twofold downregulated are represented in the lower right section with green dots (these genes are analytically listed in the adjacent table).

We performed quantitative RT-PCR analysis for 84 angiogenesis related genes in blood from patients with breast cancer and fibroadenoma before surgery. A scatter-plot analysis of the results together with a heat map is shown in Figure 3, as well as a comprehensive list of all the changes*.*

**Figure 4:**
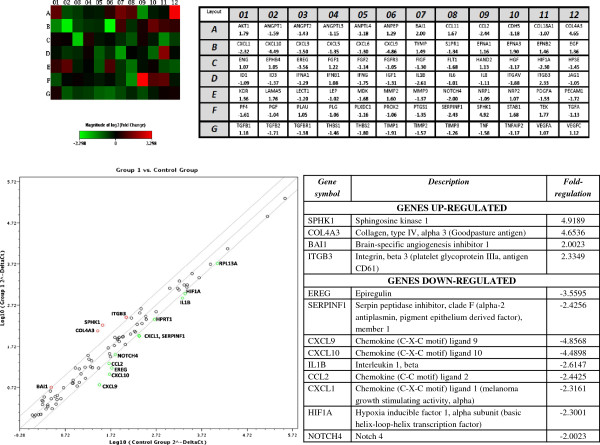
**Scatter plot analysis and heat map of all patient samples on postoperative day 3.** Significantly upregulated genes (for example, SPHK1) are depicted in a more intense red color (F09), while significantly downregulated genes are shown with a more intense green color (for example, CXCL10 in B02). The graph below plots the log_10_ of normalized gene expression levels between the control group (*x*-axis) and the breast cancer group (*y*-axis). Genes with a more than twofold upregulation in the breast cancer group are depicted in the upper left corner with red dots, while genes which are more than twofold downregulated are represented in the lower right section with green dots (these genes are analytically listed in the adjacent table).

We also analyzed the expression of gene transcripts between patients with cancer and benign breast disease after surgery in an effort to identify possible differences in expression patterns between these two groups of patients as a result of the surgical procedure itself. Results are shown in Figure 4*.*

Surgery induced an altered expression in several keygenes in breast cancer patients. We identified an upregulation of SPHK1, COL4A3, BAI1 and ITGB3, and downregulation of EREG, SERPINF1, CXCL9, CXCL10, IL1B, CCL2, CXCL1, HIF1A and NOTCH4.

### Postoperative kinetics of angiogenesis-related genes in breast cancer (postoperative day 3 to day 7)

Analysis of changes in gene expression between postoperative days 3 and 7 revealed overexpression of several circulating transcripts which were previously underexpressed*.* Moreover, COL4A3, which was initially upregulated, showed significant downregulation between days 3 and 7. CXCL9, FGF1 and TIMP3 seem to show an opposite trend.

## Discussion

We investigated the impact of mastectomy for breast cancer on circulating molecules of angiogenesis. For this purpose, serial blood measurements were obtained from 10 cancer patients who underwent mastectomy before and up to postsugery day 7, and 6 patients with fibroadenoma who served as a control group.

VEGFA is a key player in the process of angiogenesis [18]. Several studies have tried to link circulating VEGFA with disease stage and prognosis [19]. In our study, preoperative levels of this angiogenic factor were found significantly higher in the breast cancer group compared to the control group. Postoperatively, a transient increase in circulating levels of VEGFA was documented for nearly all patients who underwent surgery for breast cancer 3 days after surgery which waned back 4 days later. FGF2 is another major proangiogenic cytokine [20]. We found a trend towards increased levels in almost all patients, which has also been shown by others [15]. Finally, IL8 increased only marginally after surgery on day 3 but settled down to values near the detection limit 1 week postsurgery.

We also analyzed perioperative kinetics of circulating transcripts of angiogenesis-related genes to profile gene expression patterns. We first compared the results from the samples that were obtained preoperatively in both the control group and the cancer group. We found upregulation of a significant number of angiogenesis-specific genes in breast cancer patients compared with controls: sphingosine kinase 1 (SPHK1), epidermal growth factor (EGF), vascular endothelial growth factor C (VEGFC), neuropilin 1 (NRP1), fibroblast growth factor (FGF1), laminin alpha 5 (LAMA5), collagen type IV alpha 3 (COL4A3), IL8, ephrin B2 (EFNB2), EFNA3, tyrosine endothelial kinase (TEK), integrin beta 3 (ITGB3), V-akt murine thymoma viral oncogene homolog 1 (AKT1), thrombospondin 1 (THBS1), chemokine (C-C motif) ligand 11 (CCL11) and TIMP metallopeptidase inhibitor 3 (TIMP3). This finding can be explained by the fact that, even at early-stages of disease, the process of angiogenesis has already prompted upregulation of certain proangiogenic factors.

Some of the identified factors have an established role in the process of angiogenesis. EGF is overexpressed in triple-negative breast cancer [21] and has a synergic role to the induction of angiogenesis by VEGF [22]. VEGFC binds to its receptor VEGFR3 and promotes lymphatic hyperplasia and lymphangiogenesis [13,23,24]. FGF1 has also been shown to possess proangiogenic properties [12,25] and IL8 is another established proangiogenic cytokine [26,27]. However, the greatest amplification of genes was seen with SPHK1, EGF and VEGFC. SPHK1 is one of two (together with SPHK2) generating enzymes of sphingosine-1-phosphate (S1P), which is an active sphingolipid metabolite. During the last decade, several studies have revealed the emerging role of S1P as a key molecule in human cancer development [28], angiogenesis and lymphangiogenesis [29]. Overexpression of SPHK1 in tumors [30] is now believed to be the result of hypoxia and HIF1α induction [31]. Moreover, SPHK1 has been shown to have a prognostic significance in breast cancer [32]. On the other hand, CXCL10 (also named IP-10), which is a member of the CXC chemokine family with potent antiangiogenic properties, was found to be downregulated in breast cancer patients [33-35].

We also sought to determine the effect of surgery upon the process of angiogenesis by comparing any possible alterations in specific gene expression between the two groups of patients on postoperative day 3. SPHK1, COL4A3, BAI1 and ITGB3 demonstrated a significant upregulation, while a number of other factors (mostly chemokines) were downregulated (Figure 4). SPHK1, COL4A3 and ITGB3 continue to be upregulated, as they were preoperatively. Apart from these genes, brain-specific angiogenesis inhibitor 1 (BAI1) also displays an augmented expression. BAI1 is a factor that has been mostly studied in the context of brain tumors, but it may well possess antiangiogenic effects upon other malignancies [36]. On the other hand, a hard to explain downregulation was seen for certain circulating angiogenesis promoters, such as HIF1, NOTCH and CXCL9.

Finally, we analyzed longitudinal recordings of transcript expression data in breast cancer patients that focused on three time points: preoperative, and days 3 and 7 postoperatively. We detect an initial upregulation for COL4A3 on day 3, followed by a later downregulation towards day 7. The opposite trend was shown for CXCL9, FGF-1 and TIMP-3.

## Conclusions

The results of this study demonstrate that breast cancer patients have a different expression profile of angiogenesis-related factors when compared to patients with benign breast diseases. Moreover, we found that surgical wounding might influence the process of angiogenesis in the context of wound healing, demonstrating for the first time that a broad panel of angiogenesis-related genes shift expression patterns after surgery. A more thorough study of gene profiling could possibly offer new insights for the establishment of specific biomarkers with prognostic significance in breast cancer and potential implications in decisions regarding postsurgical adjuvant therapy.

## Consent

Written informed consent was obtained from all the patients for the publication of this report and any accompanying images.

## Abbreviations

BAI1: Brain-specific angiogenesis inhibitor 1; CCL11: Chemokine (C-C motif) ligand 11; COL4A3: Collagen type IV alpha 3; EDTA: Ethylenediaminetetraacetic acid; EFN: Ephrin; EGF: Epidermal growth factor; ELISA: Enzyme-linked immunosorbent assay; FGF2: Fibroblast growth factor 2; IL: Interleukin; ITGB3: Integrin beta 3; LAMA5: Laminin alpha 5; NRP1: Neuropilin 1; RT-PCR: Reverse transcriptase polymerase chain reaction; S1P: Sphingosine-1-phosphate; SPHK1: Sphingosine kinase 1; TEK: Tyrosine endothelial kinase; THBS1: Thrombospondin 1; TIMP3: TIMP metallopeptidase inhibitor 3; VEGFA: Vascular endothelial growth factor A; VEGFC: Vascular endothelial growth factor C.

## Competing interests

The authors declare that they have no competing interests.

## Authors’ contributions

GKG and HB performed all the surgical procedures. GKG, MI and IS performed the PCR arrays. MI, IS and KV performed the ELISA assays. GKG, EB and MF conceived the study, participated in its design and coordination and helped to draft the manuscript. All authors read and approved the final manuscript.
